# Introduction of modified blood priming technique in the neonatal and infant populations undergoing congenital heart surgery

**DOI:** 10.1051/ject/2026018

**Published:** 2026-09-15

**Authors:** Kailey Fuegmann, Kevin Charette, Amy Falconer, Navriti Sharma, Brian Perfette, Moore Phillips, Lyubomyr Bohuta

**Affiliations:** 1 Seattle Children’s Heart Center, Seattle Children’s Hospital 4800 Sand Point Way NE Seattle WA 98105 USA

**Keywords:** Modified blood prime, Retrograde autologous prime, Post-dilutional hematocrit, Cardiopulmonary bypass, Bloodless congenital heart surgery

## Abstract

*Background:* Allogenic blood product transfusion has well-documented risks and adverse outcomes. To limit perioperative transfusions for neonates and infants undergoing cardiac surgery, our institution launched a blood conservation program in May, 2021. Over the past several years, our standard has changed from blood priming the cardiopulmonary bypass (CPB) circuit for most patients ≤12 kilograms (kg) to administering an asanguinous prime to all patients with an adequate baseline hematocrit (HCT), regardless of weight. For those requiring transfusion, the technique of modified blood priming (MBP), introduced herein, presents an intermediate solution between a clear and standard blood prime, with the goal of administering the minimum amount of packed red blood cells (PRBC) to maintain safe oxygen-carrying capacity while on CPB. *Methods:* The crystalloid prime in the core of the CPB circuit (venous reservoir, arterial pump boot, and oxygenator) is displaced with a pre-calculated amount of PRBC to maintain an on-bypass HCT of ≥24%. In addition, the crystalloid in the arterial and venous lines is replaced with patient blood volume via retrograde and venous autologous priming (RAP and VAP). Once CPB is established, the remaining crystalloid in the ultrafiltration (UF) and cardioplegia (CPG) circuits is displaced into a syringe with circulating blood to minimize any further hemodilution. *Results:* We have applied MBP to 26 patients weighing less than 7 kg. The mean amount of PRBC these patients received during CPB, including in the prime, was half the amount when compared to the initial amount of PRBC we include in our institution’s standard blood prime alone. *Conclusion:* The MBP technique allows safe initiation of CPB in the subset of neonates and infants with a post-dilutional hematocrit (PDHCT) calculated at less than 24% while avoiding excessive transfusion of exogenous blood products.

## Introduction

Perioperative administration of exogenous blood products is a common practice across most centers performing neonate and infant congenital heart surgery [1–4]. While transfusion is often considered necessary, potential adverse outcomes associated with blood administration are well-documented in both adult and pediatric populations. These include increased incidence of nosocomial infection, prolonged duration of mechanical ventilation, and increased length of hospital stay [4–9]. Despite the known risks, blood management in this populace has remained relatively unchanged over the past few decades, with the majority of patients receiving large volumes of packed red blood cells (PRBC) and fresh frozen plasma (FFP) intraoperatively, often by way of the cardiopulmonary bypass (CPB) prime [1, 2]. Limited studies have been conducted to guide transfusion therapy during neonate and infant CPB, the most referenced of which points to 24% as the safe nadir hematocrit (HCT) on bypass with no shown benefit in maintaining a higher threshold of 35% or greater [10]. Moreover, in a large Society of Thoracic Surgeons database study, it was found that transfusion over 42% was associated with increased morbidity [11].

In May of 2021, our institution implemented a stepwise blood conservation program pursuing perioperative bloodless heart surgery in the neonate, infant, and pediatric populations. After optimization of the CPB circuit to reduce prime volume across all weight ranges, our program then re-evaluated our standard blood prime that consisted of 180 mL PRBC and 100 mL of FFP for patients ≤12 kilograms (kg). Our guidelines have changed from administering this blood prime to most patients within that weight range to now considering all patients, including neonates, candidates for a clear prime if the post-dilutional hematocrit (PDHCT) calculates ≥24%. In separate 2024 reports, we presented our bloodless heart surgery strategy for neonates and infants, including stat 5 surgical repairs such as the Norwood operation [12, 13]. We demonstrated that while challenging, bloodless heart surgery is achievable in this patient population. However, we also identified a subset of patients that ultimately required transfusion once on bypass. To maintain a blood-sparing approach intraoperatively, we introduced the modified blood prime (MBP) method, focusing on finding a safe middle ground between under- and over-transfusion. The following report describes our technique of priming the CPB circuit with the minimum amount of PRBC necessary to maintain adequate oxygenation status at CPB initiation.

## Materials and methods

**Figure 1:**
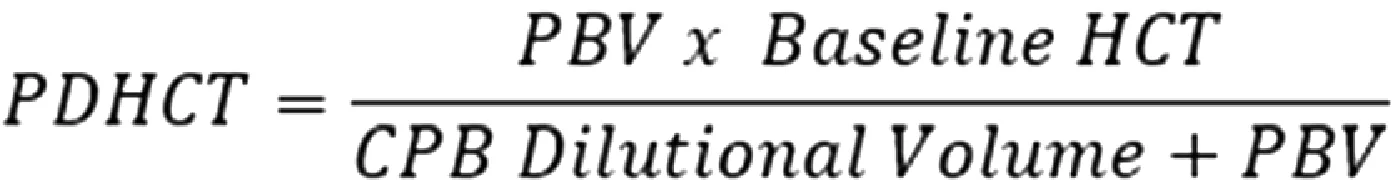
Post-dilutional hematocrit calculation. PBV is a weight-based estimated blood volume of a patient. CPB: Cardiopulmonary bypass; HCT: Hematocrit; PBV: Patient blood volume; PDHCT: Post-dilutional hematocrit.

**Figure 2:**
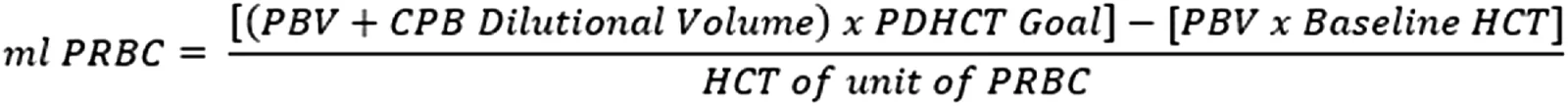
Calculation to determine the amount of PRBC to add to circuit to maintain a goal PDHCT. CPB: Cardiopulmonary bypass; PBV: Patient blood volume; PDHCT: Post-dilutional hematocrit; PRBC: Packed red blood cells.

**Figure 3:**
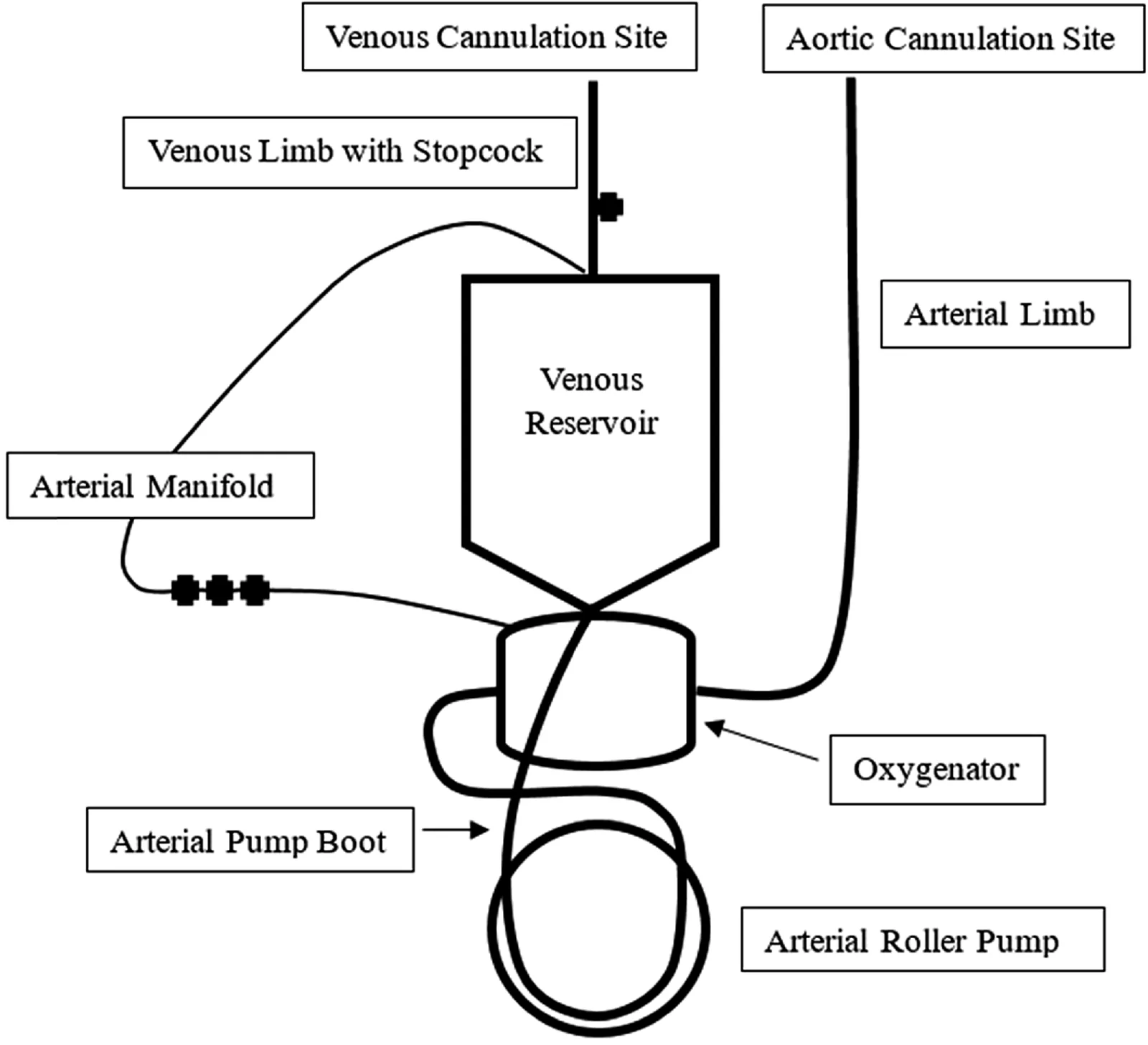
Configuration of our center’s bypass circuit.

Our neonate (≤4 kg) and infant (4–12 kg) circuits prime with 126 and 142 mL, respectively. Upon patient arrival in the operating room, an arterial blood gas is obtained, and the PDHCT is calculated (Figure 1). To accommodate the crystalloid volume removed via retrograde and venous autologous priming (RAP and VAP), when calculating the PDHCT, we use CPB dilutional volumes of 70 mL for the neonate circuit and 90 mL for the infant circuit. If the PDHCT is ≤24%, we calculate the required amount of PRBC necessary to achieve ≥24% on bypass (Figure 2). The bypass circuit is primed with a balanced crystalloid solution, sodium bicarbonate, heparin, and 25% albumin in the same manner as we would for patients receiving a clear prime. The patient is then heparinized, the aorta cannulated, and connected to the arterial line of the CPB circuit (Figure 3). After testing patency, the arterial line is clamped by the surgeon just distal to the arterial cannula to avoid mixing patient blood with the circuit clear prime. The arterial line is also clamped by the perfusionist just superior to the bubble detector for enhanced safety. The level sensor is then disabled, and any remaining volume in the reservoir is removed by turning on the arterial pump head, displacing the excess crystalloid into a waste syringe via the circuit manifold. Careful attention is paid to the circuit during this time to avoid air entrainment. To avoid mixing patient blood with clear prime, pump suction is not turned on until the reservoir is completely empty. Next, the pre-calculated amount of PRBC is dropped into the venous reservoir, and the level sensor is reactivated. Crystalloid in the pump boot and oxygenator is displaced into the waste syringe on the manifold until the level sensor is triggered and the oxygenator is partially to fully primed with the red cells. Our center uses leukocyte-reduced, irradiated blood for intraoperative transfusion of cardiac patients. PRBC are not washed via cell saver prior to administration. No FFP is introduced into the pump prime. While the perfusionist is adding blood to the circuit, the surgeon is completing the venous cannulation. Once venous cannulation is complete and the core of the circuit is primed with the calculated amount of exogenous PRBC, RAP is initiated. The arterial line clamps are removed, and the crystalloid in the arterial line is replaced with patient blood by backfilling the manifold syringe. RAP is complete when the patient’s blood fills the entire arterial line up to the outlet of the oxygenator. Finally, the crystalloid in the venous line is removed via VAP by drawing back on a syringe placed on a three-way stopcock on the venous limb just prior to entering the venous reservoir. Bypass is then initiated by removing the venous clamp and turning on the arterial pump to establish forward flow. Once stable on CPB, the ultrafiltration (UF) and cardioplegia (CPG) circuits are blood primed using the available circulating blood volume. If the venous reservoir level cannot be maintained at full flow after initiation, 25% albumin is administered in 10–20 mL aliquots. If the reservoir level cannot be maintained and the HCT drops below 24%, then 5–10 mL/kg PRBC is transfused incrementally. Indications for further transfusion on bypass include: a 20% drop in near-infrared spectroscopy (NIRS) from bypass baseline, venous oxygen saturation (SVO2) less than 60%, blood pressure unable to be maintained without excessive pharmacologic intervention, and unacceptable lactate rise. Hemoconcentration is utilized as necessary throughout the bypass run to remove any excess volume and maintain the HCT ≥24%. Patients undergo modified ultrafiltration (MUF) post-CPB to mitigate any further hemodilution.

## Safety

The CPB circuit is equipped with bubble, pressure, and level monitoring. The bubble sensor is active during the entire MBP procedure, and the level sensor is activated prior to initiation of bypass. The level sensor is placed so that the arterial pump servo-regulation is activated at the top of the suggested minimum operating level of the reservoir. The arterial line pressure is monitored during the RAP procedure to ensure there is always positive pressure in the line. Moreover, two arterial line pressure transducers are utilized, with the second set to alarm if negative pressure is seen in the arterial line. This second transducer is a safety mechanism adapted from our MUF circuit. To ensure bypass can be quickly initiated in case of hemodynamic instability, RAP is not started until after venous cannulation. During RAP and VAP, the patient’s filling and mean arterial pressures are monitored closely. Phenylephrine is administered by anesthesia in 10–50 microgram (mcg) doses to support the blood pressure if necessary. NIRS is also continuously monitored. If the NIRS values drop by more than 20% from baseline or MAP is unable to be maintained, bypass is immediately initiated.

## Results

Since November of 2023, we have utilized MBP on 26 patients (Table 1). Patients’ weights ranged from 2.0 to 6.5 kg with a baseline HCT between 21% and 31%. Patients received between 33–160 mL PRBC during the bypass run. A mean of 52 mL PRBC was administered during MBP (Table 2). A mean of 96 mL PRBC was given throughout the duration of bypass, including the amount administered in the prime. Mean post-MUF HCT was 29.8%. No patients received FFP while on bypass.

**Table 1:** Patient HCT in relation to goal PDHCT and PRBC administered throughout the CPB case. CPB: Cardiopulmonary bypass; HCT: Hematocrit; MUF: Modified ultrafiltration; PDHCT: Post-dilutional hematocrit; PRBC: Packed red blood cells.

Weight (kg)	Baseline HCT (%)	Target PDHCT (%)	Prime PRBC (mL)	PDHCT on CPB (%)	Total Blood given on CPB (mL)	Post-MUF HCT (%)
6.5	27	24	52	25	117	28
3.3	28	24	33	24	33	25
4.3	28	28	36	21	145	31
5.4	27	28	50	26	100	33
6.2	24	24	45	20	105	32
4.6	26	26	43	24	66	20
3.9	27	27	58	27	58	28
4.4	29	27	80	29	80	34
3.7	27	24	63	25	63	26
4.7	24	26	26	25	160	39
5.9	24	27	50	26	100	29
6.5	25	27	52	24	95	28
5.2	29	28	55	25	55	26
2.0	31	27	62	25	92	36
3.6	28	27	42	24	78	30
4.0	26	27	47	23	87	31
2.8	26	27	66	22	116	28
3.7	26	27	50	27	88	31
2.3	29	27	25	21	74	33
2.7	30	27	37	24	119	30
4.7	23	27	80	27	127	32
4.6	26	27	63	25	109	31
2.2	26	27	46	24	134	26
3.6	25	27	47	24	72	32
2.5	21	27	70	24	85	24
5.0	24	27	76	25	126	32

**Table 2:** Mean amount of PRBC administered in relation to the mean HCT pre-, during, and post-CPB. CPB: Cardiopulmonary bypass; HCT: Hematocrit; MUF: Modified ultrafiltration; PDHCT: Post-dilutional hematocrit; PRBC: Packed red blood cells.

Baseline HCT (%)	Prime PRBC (mL)	PDHCT on CPB (%)	Total blood given on CPB (mL)	Post-MUF HCT (%)
26.4	52	24.5	96	29.8

## Limitations

In early patients, the initial target PDHCT on CPB varied between 24 and 29%, with the value largely determined by surgeon preference and patient baseline HCT. We found that slight variations in patient estimated blood volume (EBV), HCT of exogenous PRBC, and the amount of dilutional volume administered by anesthesia prior to initiation of bypass made exact MBP calculations challenging. Moreover, determining the boundary between the clear prime and patient blood remains inexact, with mixing inevitably occurring during the process. In an effort to standardize our practice and provide a small buffer for these difficult-to-account-for variables, we set the goal PDHCT at 27% midway through the cohort.

## Discussion

The calculation to determine PDHCT is taught in all perfusion schools as standard curriculum; however, prior to establishing our institution’s blood conservation program in May, 2021, it was infrequently used in our practice. Prior to then, blood priming the bypass circuit for neonates and infants was a ubiquitous endeavor. Our neonate and infant circuits were primed with a minimum of 180–220 mL of PRBC and half a unit of FFP, followed by pre-bypass ultrafiltration (PBUF) to filter the exogenous blood. What was left of the remaining PRBC unit would mostly be infused during bypass. The protocol, at that time, was to maintain a HCT in the mid-30% while on bypass, followed by MUF post-CPB, often driving the HCT >40%. This protocol followed the logic that PRBC transfusion was mostly beneficial, coupled with the belief that wasting exogenous blood a patient was already exposed to was irresponsible. Moreover, maintaining a higher HCT into the post-bypass period was necessary due to our concurrent practice of administering platelets after CPB, which created another route of hemodilution. Patients often received upwards of 400 mL of exogenous products prior to leaving the operating room during this era.

**Figure 4:**
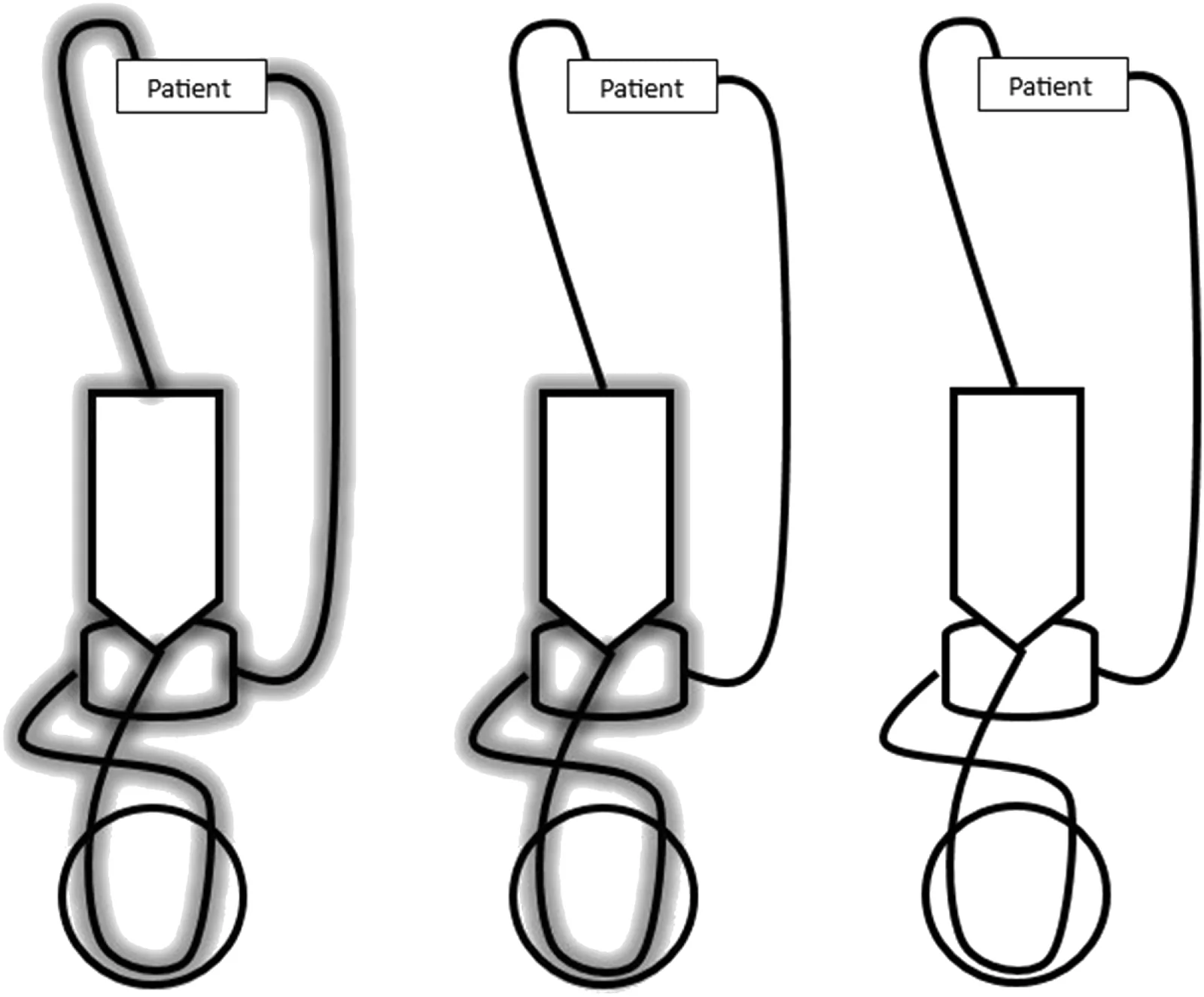
Far left diagram represents standard blood priming at our institution. Middle diagram represents MBP. Far right represents clear priming. Highlighted areas on each diagram represent areas of circuit primed with PRBC. Areas without highlight are clear primed and subject to RAP or VAP as the patient tolerates prior to initiation of CPB.

At the start of our blood conservation efforts, we incrementally implemented circuit modifications to reduce CPB prime volumes across all patient weight ranges. Notably, our infant circuit decreased from a prime volume of 200 to 142 mL, and our neonate circuit decreased from 165 to 126 mL. Secondary to these reductions, we altered our standard blood prime to include 180 mL PRBC and 100 mL FFP, filtered via PBUF prior to CPB. As we gained more experience with blood-sparing techniques, we began utilizing PDHCT routinely and started considering all patients for an asanguinous prime if calculated at ≥24%. While we found that bloodless surgery is possible in the neonate and infant populations, we recognized that it is not achievable in all patients. Focused on maintaining an intraoperative blood-sparing approach, we developed the MBP technique, an intermediate solution between a clear and standard blood prime for these patients (Figure 4). In comparison to our standard blood prime, the MBP cohort was transfused a mean amount of 96 mL PRBC throughout the entire CPB run, including the mean amount of 52 mL PRBC administered in the prime. No FFP was transfused during CPB. Additionally, no platelets or cryoprecipitate were administered in the operating room. PBUF was not required due to the low volume of exogenous blood added to the prime.

Recent literature has demonstrated that a greater total volume of blood products administered per kg was associated with a longer duration of mechanical ventilation in infants undergoing cardiac surgery [6]. Furthermore, a retrospective analysis found that in addition to the amount of product transfused, the timing of transfusion affected patient morbidity, with patients who received intraoperative transfusions having longer intensive care unit (ICU) stays and duration of ventilation when compared to patients who received postoperative or no transfusion [14].

Based on our growing experience with adequate oxygen-carrying capacity and hemostasis with MBP, combined with current literature supporting a blood-conserving approach in neonatal and infant cardiac surgery, our institution has moved to using the MBP technique as our standard of care when an asanguinous prime is not achievable. No contraindications to this technique have been identified.

## Conclusion

Blood transfusion carries inherent risk. Through a combination of autologous and blood priming, the MBP technique limits the overall amount of exogenous blood administered during CPB by an average of twofold when compared to our standard blood prime alone, while safely maintaining oxygen-carrying capacity during transition to bypass.

## Data Availability

The research data associated with this article is included within the article.
